# Interaction Signatures Stabilizing the NAD(P)-Binding Rossmann Fold: A Structure Network Approach

**DOI:** 10.1371/journal.pone.0051676

**Published:** 2012-12-17

**Authors:** Moitrayee Bhattacharyya, Roopali Upadhyay, Saraswathi Vishveshwara

**Affiliations:** Molecular Biophysics Unit, Indian Institute of Science, Bangalore, India; University of Cincinnati College of Medicine, United States of America

## Abstract

The fidelity of the folding pathways being encoded in the amino acid sequence is met with challenge in instances where proteins with no sequence homology, performing different functions and no apparent evolutionary linkage, adopt a similar fold. The problem stated otherwise is that a limited fold space is available to a repertoire of diverse sequences. The key question is what factors lead to the formation of a fold from diverse sequences. Here, with the NAD(P)-binding Rossmann fold domains as a case study and using the concepts of network theory, we have unveiled the consensus structural features that drive the formation of this fold. We have proposed a graph theoretic formalism to capture the structural details in terms of the conserved atomic interactions in global milieu, and hence extract the essential topological features from diverse sequences. A unified mathematical representation of the different structures together with a judicious concoction of several network parameters enabled us to probe into the structural features driving the adoption of the NAD(P)-binding Rossmann fold. The atomic interactions at key positions seem to be better conserved in proteins, as compared to the residues participating in these interactions. We propose a “spatial motif” and several “fold specific hot spots” that form the signature structural blueprints of the NAD(P)-binding Rossmann fold domain. Excellent agreement of our data with previous experimental and theoretical studies validates the robustness and validity of the approach. Additionally, comparison of our results with statistical coupling analysis (SCA) provides further support. The methodology proposed here is general and can be applied to similar problems of interest.

## Introduction

The relationship between protein sequence-structure and its associated function has been an oft visited subject in biological literature [1], [2]. The three-dimensional structure of proteins is proposed to be encoded in its amino acid sequence, which enables them to rapidly fold into a unique structure aptly suited for its function [3]. However, with the large amount of sequence and structural data being available in the literature, it has been realized that only a limited structural space is available to the enormous repertoire of protein sequences [4]–[7]. The structural regularities underlying the 3-dimensional organization of proteins have attracted appreciable attention [8], keeping in pace with the large number of structures being solved [9]. Two critical questions raised in this context are (a) the need for a discrete definition of the fold and (b) what are the guidelines or laws that drive the formation of a fold. It has been argued that a realistic definition of the ‘fold’ can be derived by taking into consideration their genetic and evolutionary mechanisms [10]. Furthermore, it has been proposed that the optimization in backbone packing drives the selection of a limited number of folds for a diverse sequence space [11], [12]. A recent study has shown that the protein’s core atomic interaction networks carry the ‘signature’ of the domain’s native fold [13]. Insights into the interdependence between sequence-structure-function in proteins and their divergence can be obtained from a thorough and rigorous study of the available fold space, and detection of conserved structural features for a given fold from a global perspective [1].

In general, it was proposed that homologous sequences prefer similar folds and variations at the sequence level are accompanied by divergence at the structural/functional level [14]. However, with the increasing amount of sequence and structural data, this established dogma is challenged and it has been shown that a given fold can indeed accommodate a plethora of sequences [15]–[17]. Homologous sequences often change folds or are reused to perform unrelated functions in ‘moonlighting proteins’ [1], [18]. Also there are examples of chameleon sequences which can adopt several 3-dimensional structures [2]. We also encounter proteins with similar folds despite the non-homologous sequences, different functions and apparently no evolutionary connections [15]. For example, Rossmann fold, TIM barrel and the β-immunoglobins house a large number of diverse non-homologous sequences. These simple architectures seem to be chosen by evolution. An investigation of the scaffolds, driving the formation of a fold, would enhance our understanding of protein stability and folding in general, and would allow us to rationalize why a large number of sequentially diverse proteins with different functions adopt a given structural fold.

The concept of a pre-defined underlying architecture is the key feature of protein structure networks, in contrast to the random networks, and this can be conveniently explored using the concepts of graph theory [19]. The subtle interactions at the molecular level, that holds together the structures of different proteins, also encode a structural framework which forms the basis of categorizing them into a fold. It has also been suggested that additional “structural embellishments” often contribute to an additional function [1]. Also there are evolutionary constraints on amino acid substitutions to maintain interactions within and across proteins and accessibility to the surrounding water and lipids [20]. The interdependence between the distributions of amino acids at different positions has been shown to provide useful insights into their cooperative action, both at the pairwise and at higher order correlation level through statistical coupling analysis and identification of protein ‘sectors’ [21]–[23]. Previous theoretical studies have also shown that large scale dynamics fluctuations form signatures of similar structural scaffolds [24]. Molecular dynamics simulation coupled with network analysis has recently provided insights into the structural communication fingerprints for the Ras GTPase superfamily [25]. In this study, we examine the graph theoretic parameters of the NAD(P)-binding Rossmann fold domain-specific protein structure network to derive structure based signature motifs typical of this fold and the fold-conserved pairs of interactions. We bring in the global network perspective through the structure network approach and derive a fold specific representation of the diverse set of families in our chosen dataset, which enables us to examine the general structural properties of the fold. Furthermore, we analyze the structures using several network parameters to predict the consensus set of “fold-specific hot spots” i.e., key residues and interactions responsible for maintaining the given fold. Such details not only throw light on the “skeletal” structural arrangements within a fold, but also allow us to rationalize the choice of a given fold by a set of sequences. Finally, we compare our results with the sequence based Shannon entropy values [26] and available literature to establish the robustness and validity of this generalized methodology.

In this article, we have investigated the NAD(P)-binding Rossmann fold domains superfamily. The Rossmann fold was first identified in the dinucleotide-binding proteins [27] and is one of the three most prominent folds in the Protein Data Bank (PDB). It is also the most populated α/β fold. The Rossmann fold binds a mononucleotide and is comprised of a βαβαβ motif [28]. Therefore the fold that binds dinucleotides like NAD/NADP involves two such mononucleotide binding motifs related by a pseudo two fold axis, and here the two βαβαβ motifs form a six-stranded β-sheet flanked by the helices. In this study, we examine the NAD(P)-binding Rossmann fold domains of 84 structures from 8 different protein families. In proteins with high sequence similarity and significant number of conserved residues, the sequence-structure-function relations can be probed at the sequence level [21]. In cases where common 3D structures are adopted by diverse sequences, structure/fold specific signatures can be derived from the interactions within a protein, since they are better conserved than residue positions in the sequence [20]. Thus in the case of NAD(P)-binding Rossmann fold domains, the focus will be on the common spatial interactions of residues rather than sequential conservation. The aim of this study is to identify the common interactions or global structural features driving the adoption of the common fold in the NAD(P)-binding Rossmann fold domains superfamily.

## Results

We have chosen 84 high resolution structures from 8 different families hosting the NAD(P)-binding Rossmann fold domains (Table 1, Table S1 in Supporting Information S2) and propose a graph theoretical formulation to capture the “fold conserved” interactions and hot spots in molecular details (see the Methods section for a detailed description).

**Table 1:** Summary of the dataset.

S.No	SCOP ID	Family Name	Total members	Representative member
Family 1	c.2.1.1-	Alcohol dehydrogenase-like C-terminal domain	11	1P0F
Family 2	c.2.1.2-	Tyrosine dependent oxidoreductases	13	1OOE
Family 3	c.2.1.3-	Glyceraldehyde-3 phosphate dehydrogenase-like N-terminal domain	17	1Q0Q
Family 4	c.2.1.4-	Formate/Glycerate dehydrogenases,NAD-domain	5	1PJC
Family 5	c.2.1.5-	LDH N-terminal domain like	13	1LLD
Family 6	c.2.1.6-	6-Phosphogluconate dehydrogenase-like N-terminal domain	13	2F1K
Family 7	c.2.1.7-	Aminoacid dehydrogenase-like,C-terminal domain	7	1GPJ
Family 8	c.2.1.9-	Potassium channel NAD-binding domain	5	1LSS

### Fold Specific Protein Structure Network (PSN): General Structural Properties of NAD(P)-Binding Rossmann Fold Domains

**Figure 1:**
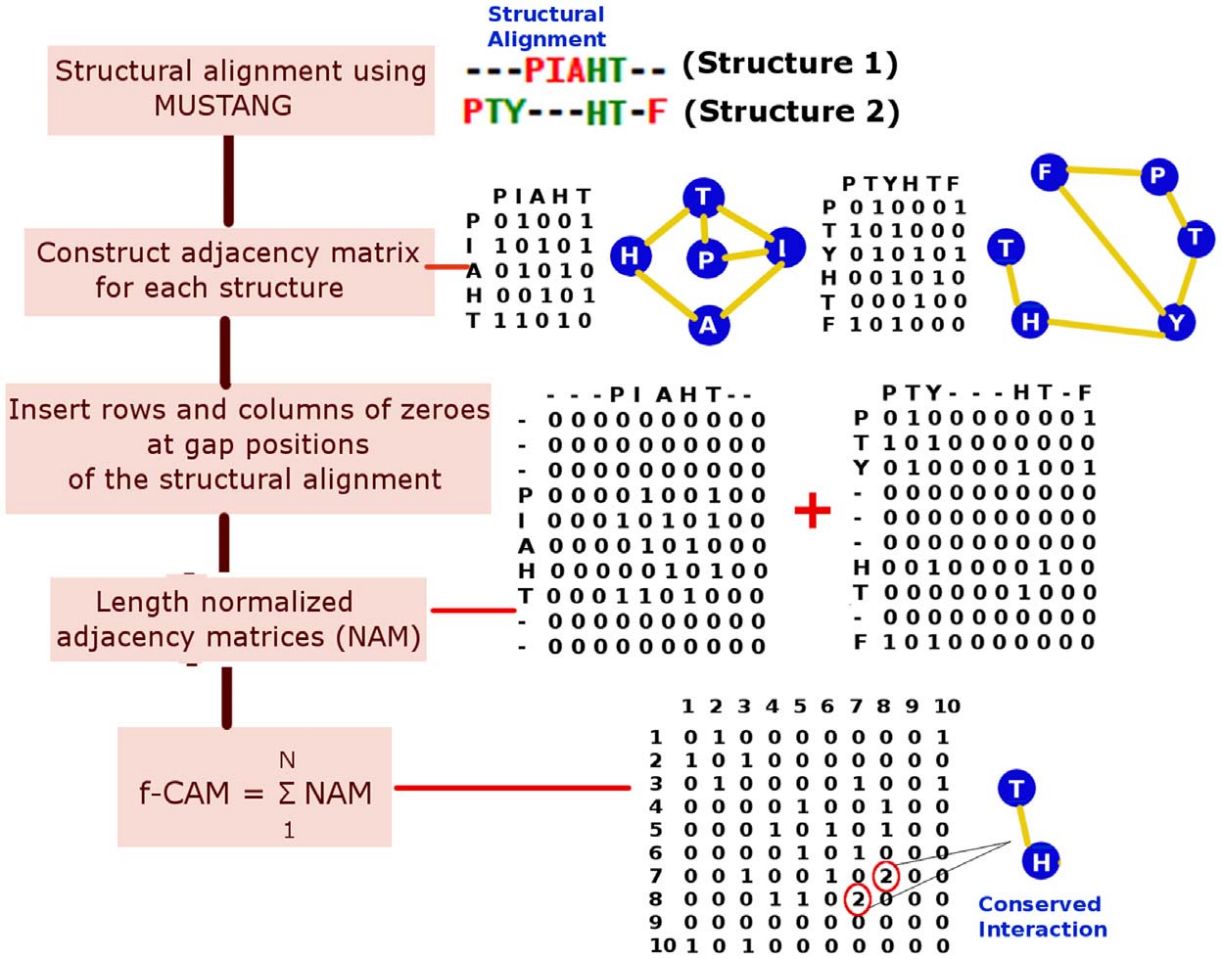
Detailed outline describing the creation of the normalized adjacency matrices (NAM) and fold-specific combined adjacency matrix (f-CAM) using a set of toy peptides PIAHT and PTYEVF. The ij^th^ position/s in the f-CAM which has hub-weight greater than 1 is highlighted in red circles. This particular interaction/edge (between position 7 and 8 in the toy example) is a conserved interaction in both the peptides.

**Figure 2:**
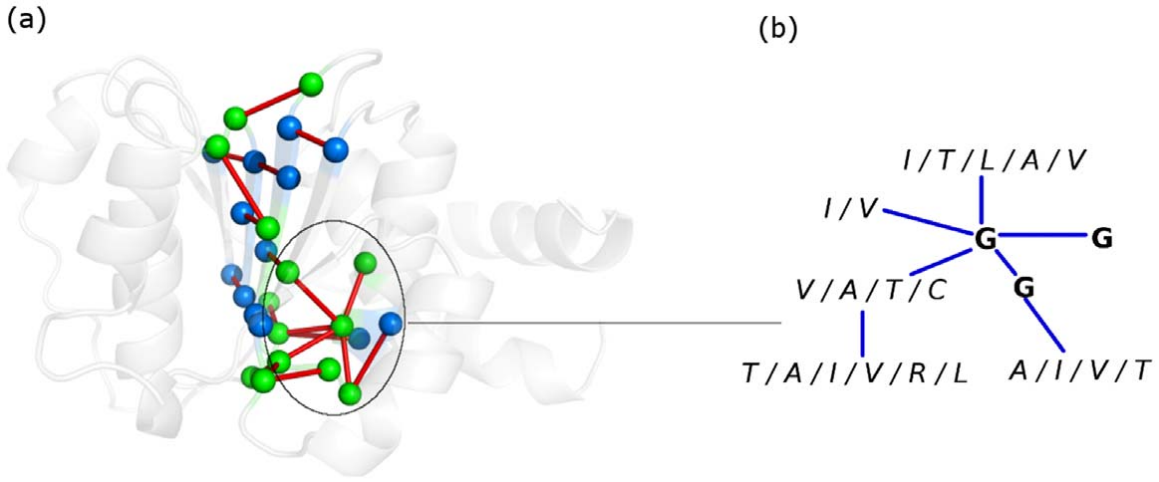
Pictorial depiction of conserved interactions across the dataset. (a) Top twenty conserved interactions/edges (indicated as red lines) in our dataset depicted on a representative member of Family 1 (PDB_id: 1P0F). The residues corresponding to the top 10 edges are colored green and those from the next 10 edges are colored blue and are represented as van der Waals’ spheres. The protein backbone is depicted as new-cartoon. A subset (the edges and the residues conserved in most of the proteins of the fold) of top 20 fold-conserved edges is highlighted in a black circle. These edges form a spatial motif for a majority of the structures in our dataset. (b) Residue participation in the spatial structural motif as obtained by considering one representative member from each family in our dataset. Two of the three Gly residues (from the conserved spatial motif) highlighted in bold are completely conserved in all the members of our dataset implying their structural importance. The Gly at the centre of the motif is a hub connecting to either hydrophobic residues or small polar residues like Thr and Cys in the selected proteins.

**Figure 3:**
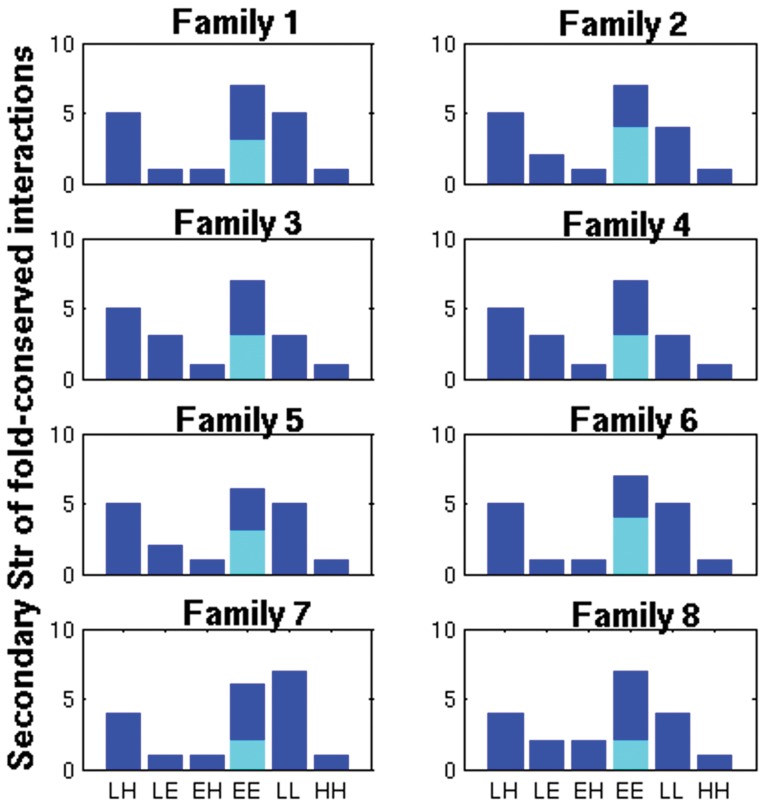
Secondary structural niche of the contributing nodes in the top 20 fold-conserved interactions/edges. The six bars on the x-axis correspond to the location of two interacting nodes making the edge (loop-helix (LH), loop-sheet (LE), sheet-helix (EH), sheet-sheet (EE), loop-loop (LL) and helix-helix (HH)).Further, the bar representing EE is split into two parts: cyan depicting the ones which are present at the middle of the strand (EE_m_) and blue depicts those formed between the termini (EE_t_) of two β-strands, respectively. It is clearly evident that there is a predominance of interactions involving the loops (L-L/H/E) and those between β-strands (EE) (EE_t_ exhibit significant contributions).

We have examined the 84 structures in our dataset using a fold specific representation (called the fold-specific Combined Adjacency Matrix (f-CAM)) of the atomic interactions, at the side chain level, in the global milieu. The f-CAM is a combined representation of the length normalized individual adjacency matrices (see Methods and Fig. 1) in the dataset (comprised of 8 different families that take up the NAD(P)-binding Rossmann fold domains according to the SCOP classification) and represents the spatially conserved interactions within the members of this fold/superfamily. The f-CAM is composed of both real (amino acid residues) and virtual (phantom nodes that are not connected to any other nodes in the network) nodes. Thus in f-CAM, each protein sequence is translated into a series of uniform positions numbers, such that every protein in the dataset can now be represented by an identical nomenclature in terms of these positions and the extent of conservation of interactions can be captured. Thus, f-CAM can provide the structural properties of the fold on the basis of conserved interactions. We consider the interactions which are present in at least half of the total number of structures in the dataset to be important for uptake of the fold by a particular sequence and subsequent stabilization. We select the top twenty interactions in f-CAM and these interactions are common to 57–83% of the total number of structures in the dataset (Table 2). These top twenty conserved patterns of interactions are pictorially depicted on a representative member (PDB_id: 1P0F) of Family 1 (Fig. 2a and a pictorial summary for representative members of the other 7 families is given in Fig. S1 in Supporting Information S1). We also probed into these conserved edges to identify the secondary structural niche of their contributing nodes and if any regular pattern is followed. This is pictorially summarized in Fig. 3 and it is clearly evident that there is a predominance of interactions involving the loops (L-L/H/E) and those between β-strands (E-E) (Table S2a–b in Supporting Information S2). However, the E-E interactions can be split into two classes: at the middle (E-E_m_) and at the termini (E-E_t_) of two β-strands, which are categorized as loops in several cases. Furthermore, the top ten conserved edges majorly involve interactions between loops or loops with helix/β-strands and such interactions seem to stitch together the various secondary structural elements in the core of the fold (Fig. 2a, Fig. S1 in Supporting Information S1). The next ten set of conserved edges involves mainly interactions between β-strands which glues together the central β-sheets in the core of the NAD(P)-binding Rossmann fold domains for each of the 8 families in the dataset (Fig. 2a, Fig. S1 in Supporting Information S1). We term these interactions as “skeletal” in keeping with their role to maintain the basic structural framework of the fold and propose that any mutations involving these residues will be detrimental to the stability of the fold.

**Table 2:** Top twenty interactions/edges in f-CAM (in terms of the positions in f-CAM) (top twenty interactions projected back on actual residue numbers for one representative member of each family is summarized in Table S2 in Supporting Tables S2).

Top 20 edges	Frequency (%)
141 151	83
147 151	78
143 194	76
151 155	76
136 414	75
133 424	73
429 561	73
141 429	70
137 151	68
141 431	68
135 425	67
134 186	65
139 193	64
425 557	64
147 152	63
136 426	59
150 429	59
428 560	58
137 427	57
141 194	57

### Spatial Structural Motifs in the NAD(P)-binding Rossmann Fold Domains

Previous studies have reported a glycine rich sequence motif (V/IXGX_1–2_GXXGXXG/A) in the NAD(P)-binding Rossmann fold domain in the loop between β-strands and α-helix and in α-helix [29]. Here we observe a consensus structural motif in all the 84 structures from 8 different families, based on non-covalent atomic interactions at the side chain level, despite their non-homologous sequences. This structural motif is composed of a cluster of aliphatic/small polar residues as depicted schematically in Fig. 2b. The constituting interactions/edges in these clusters are highly conserved across families (Table S3 in Supporting Information S2). Interestingly, this motif also seems to be Glycine rich (Gly residues are found to be sequentially conserved and also within interacting distance) and is formed of residues predominantly found in the termini/loops emanating from the termini of the central β-strands and helix (αA). Such motifs play a pivotal role in holding together the secondary structures in the core of protein. It is also worth noting that majority of conserved interactions, both at pairwise level and at the level of clusters, is dominated by the loops. This is in good agreement with previous observations that highly conserved residues occur mainly in the termini of sheets and solvent accessible loops connecting sheets with helix [30], [31]. Such an observation can be attributed to the higher flexibility (both structural and evolutionary flexibility) of the loops, and *in lieu* of this they may gain an enhanced ability to accommodate a large number of non-homologous sequences under the hood of a common fold. In a nutshell, the use of f-CAM enables us to identify the conserved patterns of interactions in the 8 different families accommodating the NAD(P)-binding Rossmann fold domains, which may be elusive from the sequence/structure based alignment studies alone.

### Network Parameters: “Fold Specific Hot Spots” in the NAD(P)-binding Rossmann Fold Domains

**Figure 4:**
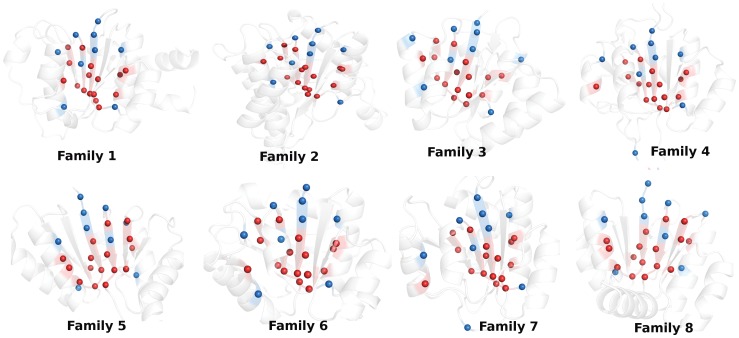
Pictorial depiction of the crucial “fold-specific residues/positions” for one representative member of each of the 8 family under study. The protein backbone is depicted as new-cartoon and these residues are represented by van der Waals’ spheres. The “fold-specific residues/positions” which are predicted to be important from all the four network parameters (region labeled 1 in Fig. 5) are colored red and those from any combination of three parameters (region labeled 2–5 in Fig. 5) are colored blue. Majority of these residues are either in the solvent exposed termini of the central β-strands or appear along the β-strands.

**Figure 5:**
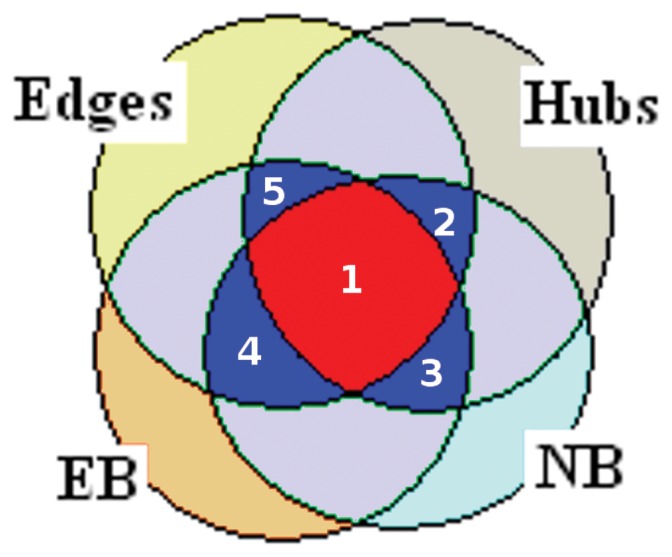
Structural conservation is measured by network parameters (Edges (pairwise interaction), Hubs (the residue with connection ≥4), NB and EB (large number shortest paths passing through the node and edge respectively)). The Venn diagram schematically shows the 13 of the 15 possible regions of overlap of the four network parameters for our dataset (PDB_ids considered are listed in Table S1 in Supporting Information S2). The red (labeled 1) and the blue regions (labeled 2–5) are either the intersection of all the four parameters or that of any three parameters and the corresponding positions/residues are considered to be fold-specific for the NAD(P)-binding Rosmann fold domains. A detail summary of the positions corresponding to the different regions of this Venn diagram is given in Table 3.

We have obtained a uniform global representation of the atomic interactions in each structure using the length Normalized Adjacency Matrix (NAM) (described in detail in the Method section). We further analyze these matrices using several network parameters like node betweenness, edge betweenness, and hubs. Hubs are important in organizing the network and maintaining its integrity by accommodating a large number of connections with other nodes/residues in the network. On the other hand, the concept of betweenness portrays the ability of a node/residue to communicate with the other residues in the network. A judicious use of these network parameters in unison and including the information of pairwise interactions from conserved edges enables us to detect a set of residues which are crucial towards the formation of the underlying framework of the protein structures. In the current context, these key residues are proposed to be the harbingers of the architecture of the particular fold (the NAD(P)-binding Rossmann fold domains in this case). We have summarized the important positions (positions provide a unified nomenclature for the residues of the different structures and can be easily projected back to a residue number), obtained from all possible combinations of the four network parameters (hubs, NB, EB, and conserved edges), for each family in our dataset in Table 3 **(** Fig. 4). The positions which are predicted to be vital from all the four network parameters and any combinations of these three (regions labeled as 1–5 in Fig. 5), are proposed to be crucial in the formation of the fold (we have pictorially depicted the residues which emulate these positions for one representative member of each family under study in Fig. 4). We propose that these positions/residues are conserved in our dataset in a fold specific manner and termed them as “fold-specific hot spots”. Strikingly, majority of these fold-specific positions are either in the solvent exposed C-termini of β-strands or appear along the β-strands for each family in our dataset (Fig. 4).

**Table 3:** “Fold-specific” important positions obtained from all possible combinations of the four network parameters (hubs, NB, EB, and conserved edges).

Hubs, NB,EB, Edges	Hubs, NB,Edges	NB, EB, Edges	Hubs, NB,EB	Hubs, Edges, EB	Hubs, NB	NB,Edges	NB, EB	Hubs, EB	Hubs, Edges	EB, Edges.	Hubs	NB	EB	Edges
136, 137, 139, 141, 151, 155, 156, 193, 425, 427, 428, 429, 531, 557, 558, 560	143, 159, 426, 536	133, 134, 135, 424	515	–	392, 660	431	559, 147,194,657	–	152, 561	–	158, 191, 399, 626, 658	508, 662	186, 414, 556	–

This table corresponds to the schematic Venn diagram (Fig. 5) showing all the possible overlaps between the four network parameters. Some of the overlap regions are empty for our current dataset and are marked in the table as ‘−’. Regions labeled 1 and 2–5 in the Venn diagram are represented by columns 1 and 2–5 respectively in the table.

Furthermore, we compute the amino acid propensities for each of these fold-specific positions from the 84 structures in our dataset. This enables us to elucidate the chemical nature of the amino acids that predominates these positions and we have pictorially summarized the amino acid propensities of the 25 fold-specific residues in Fig. S2 in Supporting Information S1. We find a prominence of aliphatic, hydrophobic residues at these positions which can be easily rationalized by their presence in mostly the core of these protein structures. However, we see an enhanced participation of charged residues at two positions, which coincide with the two fold specific residues present on the exposed loop regions of the structures (loop between β5-β4 and that between β3 and the adjoining helix). We observe a clear dominance of Gly residues for two of the fold-specific residues and it is worth noting that these Gly residues also constitute the spatial structural motif discussed in the preceding section. Also it has been previously shown that the obligatory nucleotide binding sites and nucleation/structural sites coincide in the Rossmann fold [30], [31]. So the “fold-specific hot spots” are not only ‘skeletal’ structural scaffolds, leading to the formation of the fold, but also are of functional relevance. However, some of the specific functions like dehydrogenation are performed by additional domains attached to the Rossmann fold domain.

In order to assess the robustness of the methodology at different I_min_ values, we further construct PSNs at I_min = _3 and 4%. A comparison of the residues in the context of the four network parameters (namely hubs, conserved edges, NB and EB) at I_min_ values of 2, 3 and 4% reveals good agreement (summarized in Table S4 in Supporting Information S2). Additionally we evaluated the ‘fold-specific hot spots’ at I_min_ = 3 and 4%. A large fraction of ‘fold-specific hot spots’ are overlapping at different I_min_ values (position wise details are summarized in Table S5 in Supporting Information S2), asserting the importance of these residues in maintaining the NAD(P)-binding Rossmann fold domains. It may be thus concluded that our results are independent of small changes in I_min_ values within a given range (as discussed in the Methods section), thereby establishing the robustness of the methodology.

### Higher Order Network Parameters: Cliques

We have examined higher order network parameters like cliques/communities to probe into the structural organization of the members in our dataset. We have recently shown that a comparison of cliques of non-covalent connections is a good metric to quantify structural similarity/dissimilarity [32]. In another study, we have shown that clique percolation is a unique property of a PSN [19]. We consider the residues participating in cliques at the chosen I_min_ of 2% from at least 50% of the total structures in our dataset to be important for achieving the particular structural pattern/fold. Interestingly, it is seen that 80% of the top 25 “fold-specific hot spots” coincide with the residues that participate in clique formation, indicating their influence in the higher order connectivity in the structure network (Table S6 in Supporting Information S2).

### Does Conservation in “Skeletal” Structural Interactions Follow Sequence Conservation Rules?

**Figure 6:**
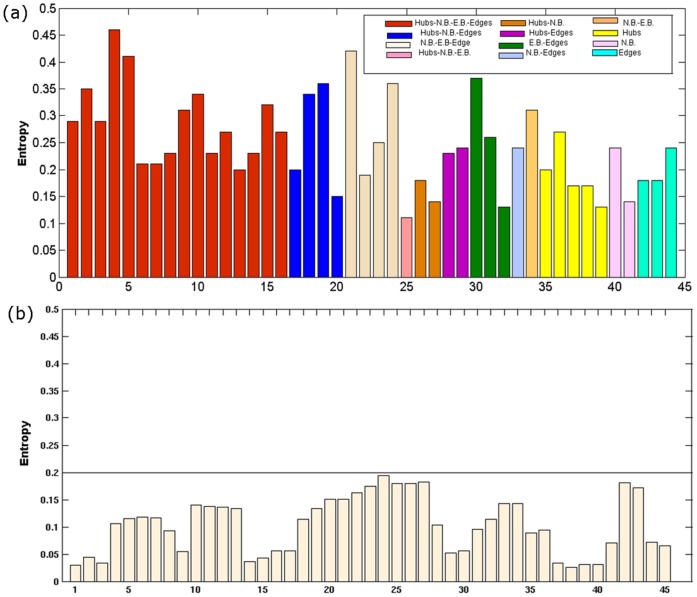
Bar plot of the Shannon entropy (SE) values. (a) Positions/residues identified as important by any combination of the four network parameters, namely hubs, NB, EB and edges exhibits SE values greater than 0.2, the maximum SE value for the dataset being 0.45 (Fig. S3b in Supporting Figures S1). (b) Positions/residues which belong to the lowest 30 positions in the hub, NB, EB and edge list clearly show SE values less than 0.2. This is a control to emphasize that the residues identified as important by the network parameters also have high SE values.

A conventional sequence or structural alignment for all the 84 structures in our dataset would yield very few completely/partially conserved residues. The calculation of Shannon entropy (SE) for each residue position is a better metric to quantify the conservation of an amino acid for a sequentially diverse dataset [26]. The highest SE value for the chosen dataset is only ∼ 0.45 (Fig. S3b in Supporting Information S1). Furthermore, the application of the graph theoretical parameters provides a comprehensive and global view of the structural organizations in the proteins and thus gives a perspective often elusive from the sequence conservation or secondary structural conservation studies. Here we obtain the SE for each “fold-specific hot spots” as identified above and it is striking that these key residues usually show better SE values (i.e. better conservation, SE values mostly >0.2) as compared to a set of control residues (SE values <0.2; the control residues being the ones identified by the network parameters in only a few structures of the dataset) (Fig. 6). A good correlation (0.75) is also obtained between the hub-weight of a node and the corresponding SE values (Fig. S3a in Supporting Information S1). These observations further validate the importance of the fold-specific residues identified using network parameters, in driving the formation of the fold. However, such a subtle level of sequence conservation (in terms of SE) would be elusive from the analysis of diverse sequences (sequence identity is summarized in Table S7 in Supporting Information S2) taking up by the NAD(P)-binding Rossmann fold. Here we discussed about sequence conservation at each site. However, correlated mutations can also provide insights into the important interactions. This is elaborated in detail in the following section.

### Comparison with Statistical Coupling Analysis and ‘Sectors’

The hierarchy of structural features underlying several biological properties (allosteric communication, folding and enzymatic function to name a few) has been extensively probed by Ranganathan’s group through statistical analysis (SCA) of the coevolved pairs of amino acids in the proteins [21], [23]. Further, it has been shown that a group of coevolved residues (‘sectors’) can be identified from positional correlations and implies evolution driven heterogeneity of interactions among amino acids, which can be ascribed to biological properties [22], [33]. Similarly, the f-CAM based network analysis segregates the proteins in terms of the heterogeneity in spatial interactions and “conservation of interactions” across the fold, facilitating the identification of structural and/or functional organizations of the fold.

**Figure 7:**
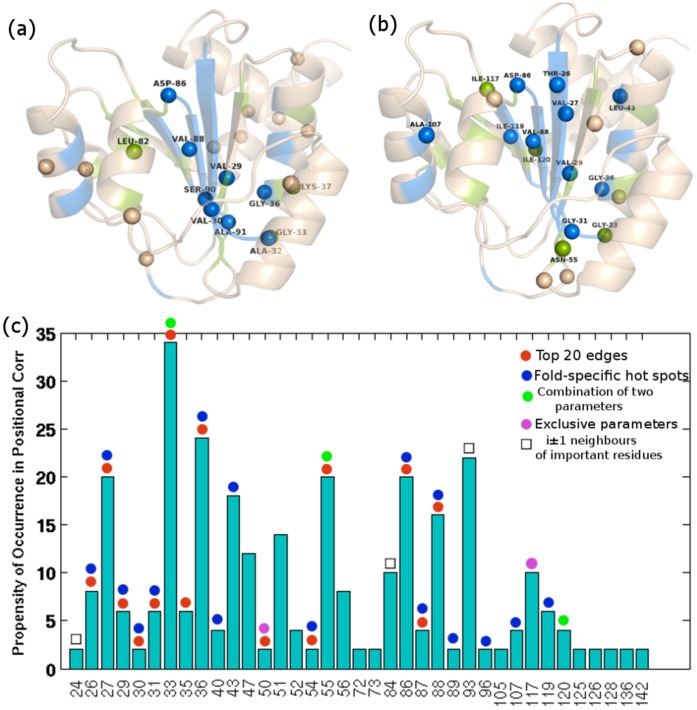
Pictorial depiction of the ‘sector’ residues on the 3D structure glutamyl-trna reductase (1GPJ). (a) ‘Sectors’ obtained from SCA on the MSA of 2373 non-homologous sequences. Out of the 23 ‘sector’ residues, 7 are also identified as important from different combinations of the four network parameters. (b) ‘Sectors’ obtained from SCA on the MUSTANG structural alignment of 84 structures. Out of the 20 ‘sector’ residues, 15 are also identified as important from different combinations of the four network parameters. The overlapping residues between SCA and network based analysis are depicted as blue and green spheres respectively and are also labeled. The backbone is shown in transparent cartoon representation with “fold-specific hot spots” colored blue and those appearing to be important from hubs, EB, NB and top edges or any of their binary combinations colored green. (c) Propensity of occurrence of residues in coevolved pairs from SCA on the structural alignment of 84 structures. Overlap of these residues with those forming top 20 fold conserved edges from f-CAM or any combination of the four network parameters are highlighted as colored dots respectively. The red and the blue dot highlights overlap with top 20 conserved edges and ‘fold-specific hot spots’ respectively. Whereas the green signifies overlap with the residues identified as important from a combination of any two network parameters, the pink dot highlights the overlap with those that are identified as important from a single network parameter. The residues overlapping with i±1 neighbors of important residues from network approach are marked with a black square. It is noteworthy that most of the SCA coevolved residues (both from sequence alignment based SCA and structure alignment based SCA) also figure out as important from the four network parameters.

Upon comparison of the ‘sector’ residues, derived from SCA of a large number of non-homologous sequences (see Methods), with the important residues identified from various combinations of the four network parameters considered, we come across a significant agreement (Fig. 7a). Furthermore, the SCA results from a structural alignment of 84 structures (also used for network analysis; Table S1 in Supporting Information S2) shows improved correlation with our data from the network analysis on f-CAM, highlighting the importance of introducing structural features to the input alignment for SCA (Fig. 7b, c) (the high correlation between the two methods may be due to the overall uniformity at the supersecondary structural level for the Rossmann fold and other folds with more diversity at the level of supersecondary structural organization may exhibit a reduced correlation). There is a noteworthy correlation, both at the pairwise interaction level (coevolved pairs from SCA and top 20 edges from f-CAM) (Fig. 7c) and at the level of collective interactions (‘sector’ residues from SCA and important residues identified from various combinations of four network parameters, namely hubs, edges, EB and NB) (Fig. 7b). The significant overlap between the important residues identified for the NAD(P)-binding Rossmann fold domains from SCA and f-CAM based network analysis that are, in principle, two entirely different approaches, furthers establishes the robustness of our methodology.

### Comparisons with Previous Experimental and Theoretical Studies

**Figure 8:**
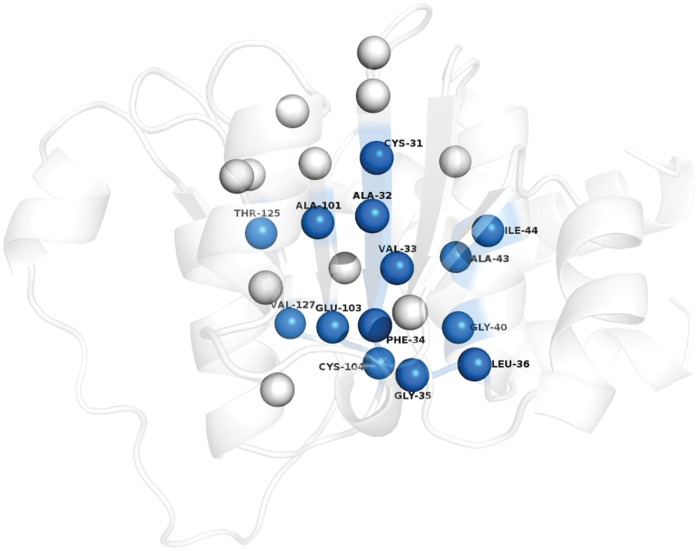
Pictorial depiction of the “fold-specific hot spots” on a representative member of Family 1. The residues are depicted as van der Waals’ spheres and the colored ones stand for the residues which have been found to be structurally and functionally important for the Rossmann fold by previous experimental and theoretical studies on CheY. A detailed residue wise comparison for a representative member from each of the 8 families in our dataset is presented in Table 4.

Previous studies based on rigorous protein engineering experiments in CheY have shown that a few residues, majorly appearing at the termini of the central β-sheets and along the β-sheets holds the key to the folding kinetics as well as catalytic function of the Rossmann fold [31]. Also, Shakhnovich's group has used the concept of ‘conservatism-of-conservatism’ (CoC) based on an alignment of intrafamily conservatism profiles to predict the “universally conserved positions in protein folds”, including the Rossmann fold with CheY as the representative structure [30]. These predicted residues with high CoC values are in good agreement with previous mutation experiments. It is remarkable that our analysis based on non-covalent spatial interactions probed through graph theory has indeed yielded results which are in excellent concordance with that of previous studies, with the identified key residues running along the β-stands and their termini as seen from earlier studies on CheY. We compare our results with those from the experiments mentioned above (summarized in Table 4) by performing a structural alignment of CheY with representative members of each family in our dataset (Fig. S4 in Supporting Information S1). Many of the “fold-specific hot spots”, identified from a combination of various network parameters (the position numbers in the NAM and the corresponding residues from one representative member of each family and CheY are summarized in Table 4) have been proposed to be important for folding kinetics of the Rossmann fold from both theoretical and protein engineering experiments. Furthermore, positions like 135, 151, and 560 are analogous to residues F7, M16, and A87 in CheY which are also predicted to be critical based on their CoC values. A87 in CheY has been proposed to be responsible for terminating β5 and a similar role can be hypothesized for all the residues at position 560 in our dataset, due to its location at the β5 strand termini (Fig. 3, 4). A pictorial depiction of the actual residues corresponding to these important positions on one representative member (1P0F) of our dataset is shown in Fig. 8. It was also proposed earlier that a ‘super-site’ with functional residues is present at the termini of the β-strands in the Rossmann fold and strikingly many of these residues are also involved in regulating the folding kinetics of the fold [30]. Thus, our observation that a majority of the “fold-specific hot spots” prefers the termini or run along the length of the central β-strands for each superfamily in our dataset is also in accordance with previous studies. The fact that most of the proteins hosting the NAD(P)-binding Rossmann fold domains are enzymes by nature, the overlap of the folding nucleation and functional sites may be an evolutionary choice to confer maximum sequestration to the enzymatic active site from external perturbations. Thus our network analysis based on the non-covalent interactions at the atomic level not only captures the residues/positions involved in driving the folding kinetics of the Rossmann fold but also predicts functionally important residues.

**Table 4:** “Fold-specific hot spot” residues obtained from a combination of all four and either of the three network parameters (derived from “Fold-specific hot spot” positions) for one representative member of each family.

Positions	CheY	Family1	Family2	Family3	Family4	Family5	Family6	Family7	Family8
**136**	**L8**	**A32**	**I6**	**T5**	**V36**	**A5**	**G4**	**L28**	**I4**
**137**	**V9**	**V33**	**V7**	**I6**	**I37**	**V6**	**V5**	**V29**	**I5**
**139**	**V10**	**F34**	**Y8**	**L7**	**L38**	**I7**	**V6**	**V30**	**A6**
**141**	**D11**	**G35**	**G9**	**G8**	**G39**	**G8**	**G7**	**G31**	**G7**
**151**	**R17**	**G40**	**G15**	**G14**	**G44**	**G13**	**G12**	**G36**	**G12**
**155**	**V20**	**A43**	**I18**	**T17**	**A47**	**L16**	**L15**	**V39**	**L15**
**156**	**R21**	**I44**	**L19**	**L18**	**A48**	**A17**	**A16**	**A40**	**A16**
193	A35	V58	I32	V34	F61	E32	V29	A54	I29
425	F52	Y100	G74	Q96	L98	M72	I60	V87	M68
427	I54	V102	F76	M98	I100	V74	F62	V89	I70
**428**	**S55**	**E103**	**C77**	**A99**	**G101**	**I75**	**I63**	**S90**	**A71**
**429**	**D56**	**C104**	**V78**	**A100**	**A102**	**T76**	**C64**	**A91**	**V72**
531	I71	A114	A114	A113	L118	L105	L77	D103	A86
557	P81	V124	L123	T119	V127	I113	I86	L118	K93
**558**	**V82**	**T125**	**L124**	**I120**	**I128**	**Y114**	**V87**	**I119**	**T94**
560	M84	V127	L126	L122	V129	L116	D89	D121	A96
**143**	**-**	**L36**	**K11**	**T10**	**G40**	**A9**	**L8**	**A32**	**I8**
159	L24	C47	F22	R22	A51	A20	L19	L43	L19
**426**	**V53**	**A101**	**V75**	**V97**	**L99**	**V73**	**I61**	**V88**	**Y69**
536	G75	T118	L118	A116	M122	A109	L81	A107	G90
424	G51	D99	D73	D95	D97	D71	K59	D86	D67
133	L5	S29	G3	K2	G33	T2	M1	K25	M1
**134**	**K6**	**T30**	**K4**	**Q3**	**K34**	**K3**	**K2**	**T26**	**Y2**
**135**	**F7**	**C31**	**V5**	**L4**	**V35**	**L4**	**I3**	**V27**	**I3**
515	L67	T110	A110	P109	R109	I101	T73	H96	S82

The residues which have been found to be important from previous experimental and theoretical studies are highlighted as bold.

## Discussion

In summary, the adaptation of limited number of folds by a large number of non-homologous sequences seems to be guided by basic physico-chemical principles. Here we have explored the structural preferences of a sequence from the perspective of global non-covalent spatial interactions using graph theoretic approach. We show that it is the universal structural architecture of the fold, held together by specific atomic interactions among residues that are better conserved, and not necessarily the residues themselves. This idea underscores the observation of a restricted fold space in contrast to the highly diverse sequence space. From our studies, we predict a number of “fold-specific hot spots” across the NAD(P)-binding Rossmann fold domains, in spite of sequence variations in the dataset. We propose that these position specific residues are crucial for the formation of the fold or in other words, offer the nucleation sites for folding. Furthermore, they are also critical for function since the nucleation and functional sites coincide for the Rossmann fold. Our results are in excellent agreement with previous experimental and theoretical studies on the Rossmann fold, in terms of the important residues identified. Additionally, our SCA results on the NAD(P)-binding Rossmann fold containing proteins exhibits a significant overlap with those derived from f-CAM based network analysis. Thus a judicious use of network theory and deft manipulation of the adjacency matrices representing the interactions in protein structure networks can provide detail insights into the structural constraints that favor formation of a common fold. A slightly modified version of this methodology in which the interactions are specified in terms of energy, has also been successfully applied recently to identify the family-specific features in the context of the TIM barrel fold [34]. This methodology can be further extended across different superfamilies, with higher sequence diversity and different functions but sharing a common fold.

## Methods

### Creation of the Dataset

The NAD(P)-binding Rossmann fold domains (SCOP_id: 51734 (fold) and 51735 (superfamily)), as classified in Structural Classification of Proteins [35], contains 12 families. The coordinates of the NAD(P)-binding Rossmann fold domains for all these structures are obtained from ASTRAL in the PDB format [36]. Culling of this full dataset is done using the *Pisces* server (http://dunbrack.fccc.edu/PISCES.php) [37] to obtain high resolution (<2Å) structures with good R-factor values (<0.25). We further chose only those families which had at least more than three structures available after the culling of the dataset. This finally produced 84 high resolution structures from 8 different families hosting the NAD(P)-binding Rossmann fold domain (summarized in Table 1 and Table S1 in Supporting Information S2) with an average sequence identity of 38.8% (std dev = 20.85; summarized in Table S7 in Supporting Information S2 and Fig. S5 in Supporting Information S1).

### Construction of Protein Structure Networks: Fold-specific Combined Adjacency Matrix (f-CAM) and Normalized Adjacency Matrix (NAM)

Protein Structure Network/Protein Structure Graph (PSN/PSG) efficiently portrays the non-covalent side-chain interactions from a global perspective [38], [39]. The details of the construction of such a graph at a particular interaction cut-off (I_min_) and the implications of such graphs have been previously discussed in detail [32], [40]. Protein structure networks are constructed by considering amino acid residues as *nodes* and *edges* are constructed between the nodes on the basis of non-covalent interactions between them (as evaluated from the normalized number of contacts between them) for each system. The non-covalent interaction between side chain atoms of amino acid residues (with the exception of Gly where C_α_ atom) are considered, ignoring the interaction between sequence neighbors. The interaction between two residues i and j has been quantified previously in our lab as:
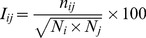
(1)where n_ij_ is number of distinct atom pairs between the side chains of amino acid residues i and j, which come within a distance of 4.5Å and N_i_ and N_j_ are the normalization factors for residues i and j. The pair of amino acid residues having interaction strength (I_ij_) greater than a user-defined cut-off (I_min_) are connected by edges to give a protein structure network (PSN) for a given interaction strength I_min_. Generally, I_min_s in the PSNs vary from 1% to 15%. We construct an adjacency matrix (a mathematical representation of the PSN) based on the non-covalent side-chain interactions at a given cut-off for I_min_, which is 2% in this study.

I_min_ is a measure of the extent of connectivity in the PSNs. A lower I_min_ is associated with higher connectivity and *vice versa*. Several previous reports from our group have shown that the optimal interaction strength in a protein structure is exhibited at an I_min_ at which the size of the largest non-covalently connected cluster (LClu) undergoes a transition. Earlier studies have pointed out that a transition in the size of the LClu is noted between an I_min_ of 2–5%. Additionally, largest community (an assemblage of cliques as discussed below) profile (LComm) as a function of different I_min_ values also indicated a transition in the I_min_ range of about 2–4%. At lower I_min_ values (pre-transition region), the network is too densely connected, whereas at higher I_min_ values (post-transition region) the network is very sparse, marking the two extremes of the I_min_ range. As a consequence, the transition regions in LClu and LComm profiles have been shown to highlight the meaningful connections in the network. Thus all our further investigations are mainly focused on the I_min_ range 2–5%, emphasizing the results at I_min_ = 2%. A comparative study at I_min_ values of 3 and 4% also illustrates outcomes that are in qualitative agreement with the results at I_min_ = 2%.

The size of the adjacency matrix is equal to the total number of residues in a protein structure. However, in order to have a normalized combined mathematical representation of all structures in our dataset (i.e. Rossmann fold specific representation), we introduce the concept of fold-specific Combined Adjacency Matrix (f-CAM) as detailed schematically in Fig. 1. A structural alignment for all the 84 structures in our dataset is done using MUSTANG [41]. The length of the individual sequences corresponding to the 84 structures is 969 after including the gap positions used for the structural alignment. The adjacency matrices, obtained as described above, for the 84 structures are then subjected to normalization by inserting rows and columns of zero (i.e., phantom residues) at the gap positions. This ensures the absence of any non-covalent interactions of these phantom “gap” residues with the other residues in the protein, thus not perturbing the actual interactions in a structure. These length normalized adjacency matrices (NAM) not only portray the connectivity information in the protein structure network, but also the positions/residues in NAM can be compared across structures. We add these NAMs to get a fold-specific combined adjacency matrix (f-CAM), in which each edge (ij^th^ position in the f-CAM) is weighed by the frequency of occurrence of that particular edge across the dataset. Thus f-CAM is a fold-specific mathematical representation of pairwise connectivity in the structures. These NAMs and f-CAM can be further probed using several network parameters to gain knowledge of the fold-specific features in general. The term positions and residues are used interchangeably in the context of NAMs and f-CAM throughout the text as these positions can be easily mapped back to actual residues in the structures taking into account the phantom residues, imitating the gap positions in the alignment (a detailed description is given in Fig. 1).

### Network Parameters: Hubs, Node and Edge betweenness

While the pairwise interactions are evaluated as an edge, the network parameters such as hubs, node betweenness and edge betweenness represent the higher order connectivity and measure of the node/edge in the global context of the protein topology. These parameters are evaluated from the NAM. A brief description of each parameter and its general physical significance in terms of the network is as follows.

- Hubs

At a given I_min_, different nodes have different number of connectivity (i.e. edges) with its neighbours. The degree distribution of the PSNs at Imin values = 2, 3, and 4% for each structure in our dataset (Fig. S6 in Supporting Information S11) clearly shows that the number of nodes with degree 4 and above drastically reduces as compared to those with degrees lower than 4. This is due to steric constraints in protein structures. Hence we have defined the nodes with four or more connections as hubs.

- Node betweenness (NB)

The term “node betweenness” represents the position of a node in the context of global topology. The communication between any pair of residues in the structure is evaluated by the parameter - shortest path (SP). Node betweenness (NB) of a node ‘i’ is defined as the number of SPs between all pairs of nodes passing through ‘i’ (Eqn. 2a). The nodes with high node betweenness (NB) represent residues which are most crucial to information flow in the network.
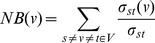
(2a)where ‘σst’ is the total number of SPs, and ‘σst(v)’ is the number of SPs passing through a node v.

- Edge betweenness(EB)

EB is the total number of SPs that pass through an edge (Eqn. 2b). Similar to NB, EB represent edges that control information transmission in the network.



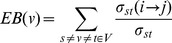
(2b)Where ‘σst’ is the total number of SPs, and ‘σst(i -> j)’ is the number of SPs passing through an edge ij.

For each NAM, the hubs residues/positions are identified and the frequency of these positions being a hub (hub-weight) in all the 84 structures is also computed. This frequency provides a quantitative measure for evaluating the importance of a position/residue in accommodating large number of interactions across the Rossmann fold and those which occur in more than half of the structures are considered to be ‘fold-specific hub residues’. A cut-off of 50% for hub-weight is optimal for our dataset. While identifying the ‘fold-specific hub residues’, we wanted to highlight only those that are present in at least half of the total structures in our dataset to ensure their importance in the context of the fold. Below this we will encounter a very large number of ‘fold-specific hub residues’, whereas at an elevated cut-off we find very few such residues, due to the high sequence diversity of the chosen dataset. The cut-off of 50% for ‘fold-specific hub residues’ and the selection of NB and EB as important parameters based on the criteria given here have been found to be optimal for the type of dataset we have currently chosen, however these criteria can be reviewed depending on factors like the number of chosen sequences, their diversity and the sequence length. Additionally, the NB and EB values are also computed for every 969 positions of each NAM. Positions with top 20 highest NB and EB are recognized for each NAM. The frequency of these high NB and EB positions in the top 20 list for each of the 84 structures is obtained and those which occur in the top 30 positions of the frequency list are considered as significant. Furthermore, the ‘fold-conserved pairwise interactions’ are also evident from the f-CAM as described in the preceding section. Finally, we combine the results from the top 30 most frequent positions of these four network parameters to predict a set of consensus critical fold-specific residues for NAD(P)-binding Rossmann fold.

### Higher Order Network Parameters: Cliques

Cliques/communities represent tightly connected regions of the network. In the context of PSN, these parameters are used to identify the rigid regions in the protein structures and to recognize the ligand induced conformational changes [32], [38]. A k-clique is defined as a set of k nodes (points represented by amino acids) in which each node is connected to all the other nodes. A *k*-clique community is defined as an amalgamation of smaller *k*-cliques that share node(s). The clique search is based on the algorithm proposed by Palla *et al.* [42]. Cfinder is used to obtain the cliques/communities from NAMs. We have used only the k = 3 cliques for our analysis (higher order cliques are rare for PSNs including the side-chain interactions).

### Amino Acid Propensities and Calculation of Shannon Entropy

The propensity of an amino acid at an identified fold-specific position of NAM is calculated from all the 84 structures in our dataset to highlight the predominance, if any, of a particular amino acid at a given position. This feature is captured by Shannon entropy (SE) for each position, which is evaluated using the CompBio server at http://compbio.cs.princeton.edu/conservation/ [26]. In this server, the base for the logarithm in the SE calculation is selected in such a way that the scores will range from zero to one, with zero indicating the highest conservation. This value is then subtracted from one. Thus a higher value of SE is an indicative of better conservation. The Mustang structural alignment is used for the SE computation.

### Statistical Coupling Analysis (SCA) and Identification of ‘Sectors’

The results from our structure network based analysis are compared with those derived from SCA and ‘sector’ analysis. The details of the SCA and ‘sector’ analysis have been described elsewhere [21], [22]. The calculations are performed using the Matlab toolbox (SCA 5.0 available at https://ais.swmed.edu/rrlabs/register.htm). 2373 non-homologous sequences from SwissProt (sequence identity <50% and sequence length <500), representing 8 protein families (Table 1) in the NAD(P)-binding Rossmann fold, are chosen for the analysis. Multiple sequence alignment (MSA) of these 2373 sequences from ClustalW is used for SCA (positions with less than 30% gaps are considered) and ‘sector’ residues are identified. Further, a separate SCA and ‘sector’ analysis is also performed on the structural alignment (using MUSTANG) of the 84 structures (summarized in Table S1 in Supporting Information S2), again after truncating the alignment to positions with less than 30% gaps. All the results are mapped back on a particular structure, 1GPJ (glutamyl-trna reductase), for ease of 3D-structural visualization.

### Comparison with Previous Experimental and Theoretical Studies

In order to compare our theoretical predictions with experiments, literature survey showed that extensive experimental and theoretical studies have focused on elucidating universally conserved residues in the Rossmann fold using a representative protein, CheY [30], [31]. However, CheY has been classified as flavedoxin-like fold in SCOP, due to a second domain and is not included by us in the construction of NAM and f-CAM. Hence we have obtained a structural alignment of the Rossmann fold domain of CheY (using MUSTANG) with one representative member of each of the 8 families of the dataset under consideration. This provides a common ground for comparing our results, by mapping back on those obtained for CheY from experimental and theoretical investigations.

## Supporting Information

Supporting Information S1 **This supporting file contains Figures S1–S6.**
(DOC)

Supporting Information S2 **This supporting file contains Tables S1–S7.**
(DOC)
