# Supplementary material for: Interaction Signatures Stabilizing the NAD(P)-Binding Rossmann Fold: A Structure Network Approach
Source: PLoS One. 2012 Dec 17;7(12):e51676. doi: 10.1371/journal.pone.0051676 (PMC3524241; doi:10.1371/journal.pone.0051676)
Supplement: Supporting Information S1 — This supporting file contains Figures S1–S6. (DOC) [file pone.0051676.s001.doc]

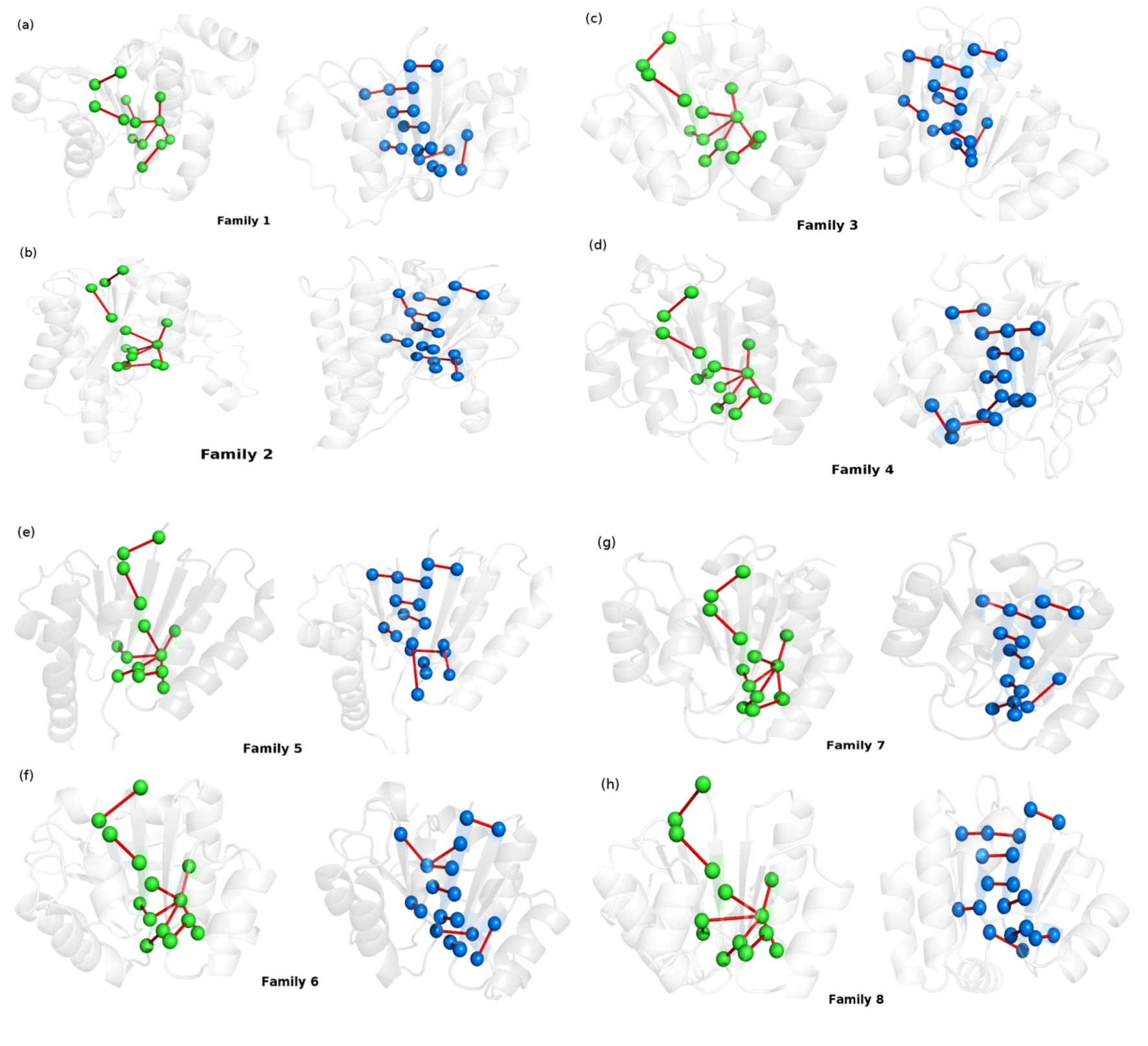


**Fig. S1**: Top twenty conserved interactions/edges in our dataset depicted on a representative member of each of the eight families in our dataset (a-h). The residues corresponding to the top 10 edges are colored green and those from the next 10 edges are colored blue and are represented as van der Waals’ spheres for each family. The protein backbone is depicted as new-cartoon. The motif highlighted in Fig3a is observed in the top 10 conserved edges in each family.


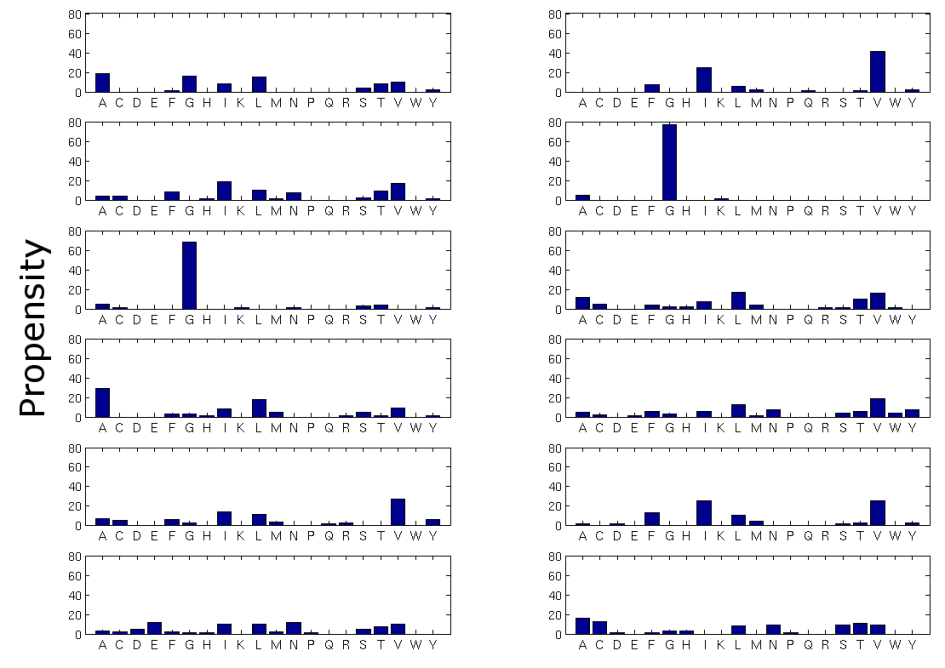


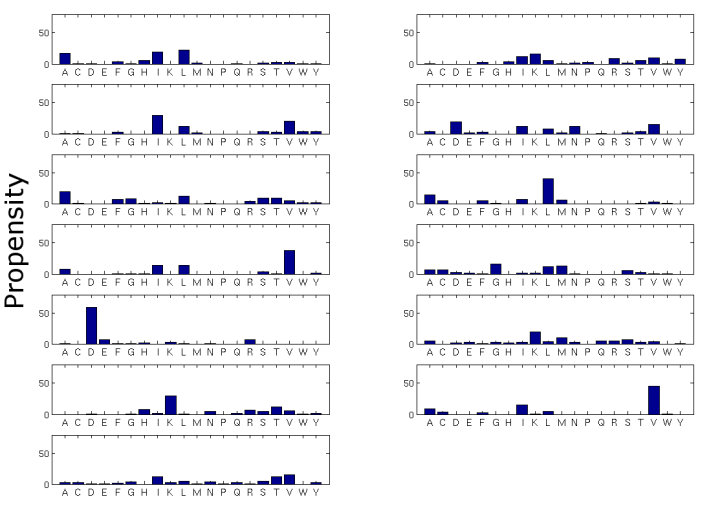


**Fig. S2**: Amino acid propensity at the top 25 “fold-specific positions”. These positions are dominated by hydrophobic residues with the exception of two positions dominated by Asp and Lys.


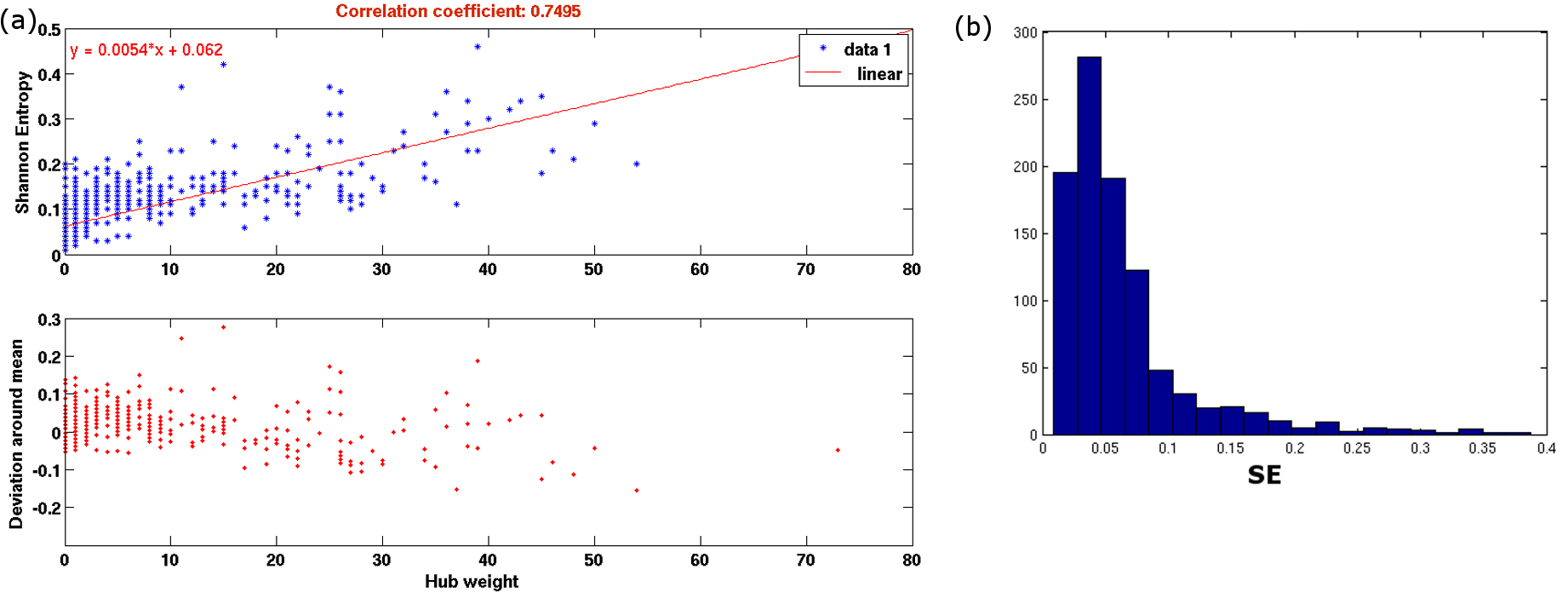


**Fig. S3**: (a) Correlation plot between the hub-weight of a node and the corresponding SE values (top panel). A scatter plot is shown in the lower panel. (b) Histogram of SE values obtained for the studied dataset [Table 1]. The highest value of SE is around 0.45 indicating the sequence diversity of the chosen dataset.


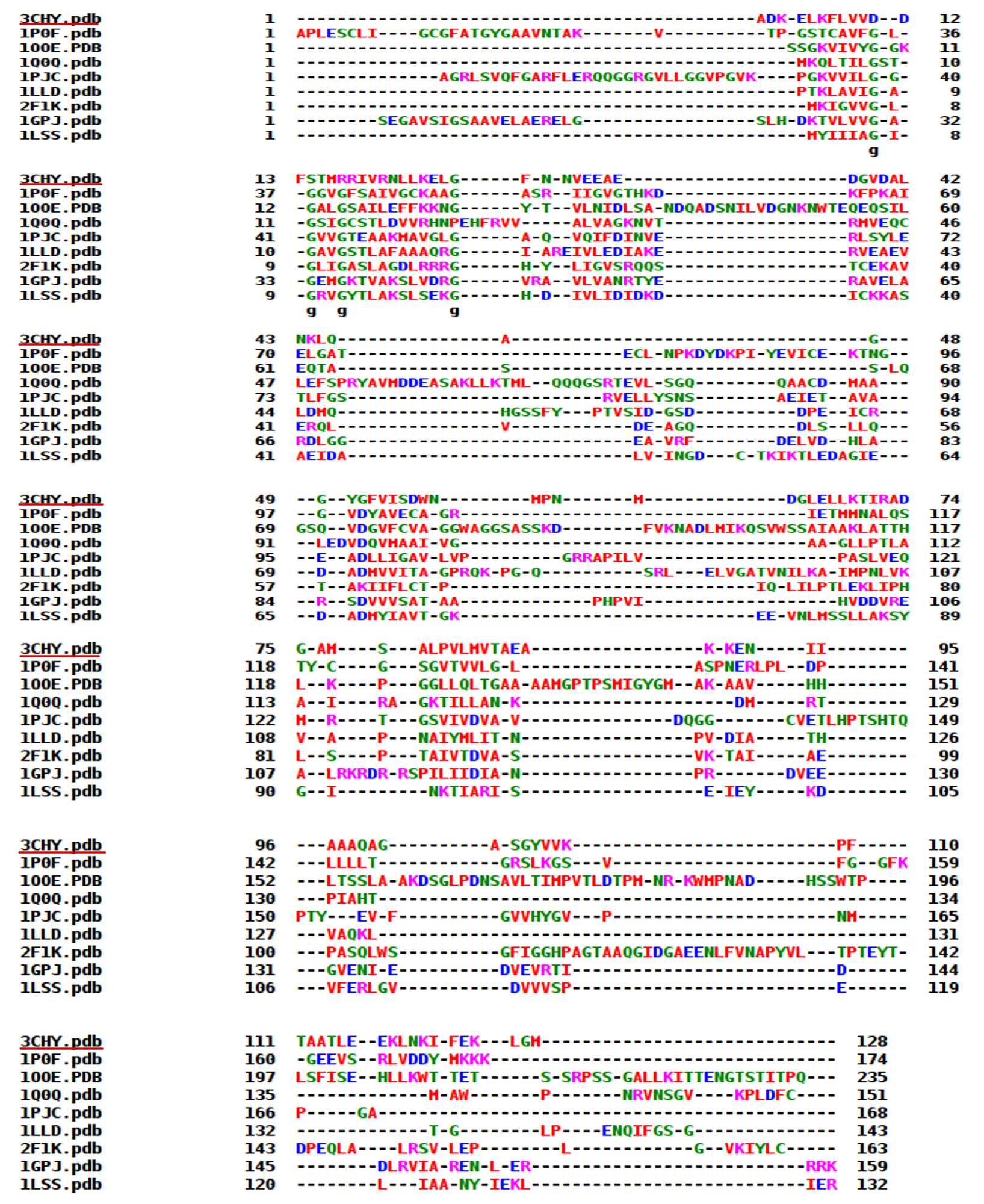


**Fig. S4**: Multiple structural alignment of CheY (PDB_id: 3CHY) with one representative member of each family (Table 1) using Mustang.


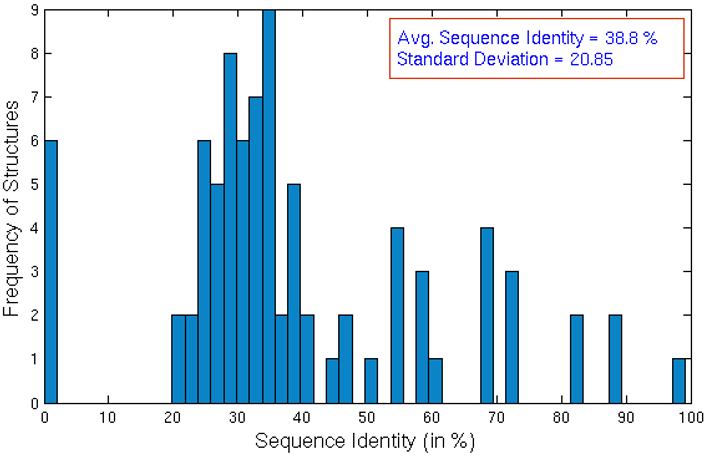


**Fig. S5**: Histogram of the maximum sequence identity of each member of our dataset with the rest of the dataset. This gives an idea of the distribution of sequence identity across our dataset of 84 structures.


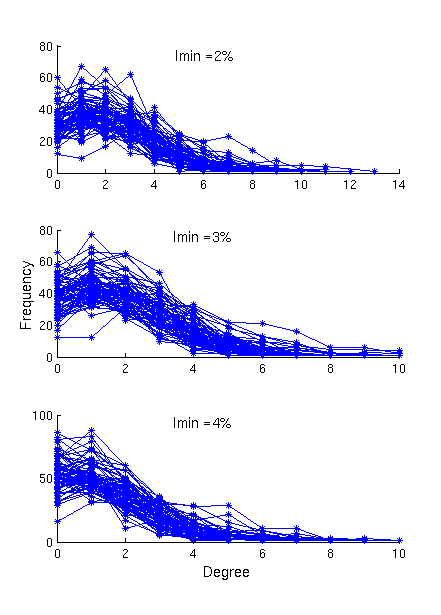


**Fig. S6**: Degree distribution for the 84 structures in the dataset at Imin values of 2%, 3% and 4%. The region between degrees 3 to 4 marks a transition from nodes with low degree and those with very high degree.
