# Supplementary material for: Interaction Signatures Stabilizing the NAD(P)-Binding Rossmann Fold: A Structure Network Approach
Source: PLoS One. 2012 Dec 17;7(12):e51676. doi: 10.1371/journal.pone.0051676 (PMC3524241; doi:10.1371/journal.pone.0051676)
Supplement: Supporting Information S2 — This supporting file contains Tables S1–S7. (DOC) [file pone.0051676.s002.doc]

**Table S1:** Summary of the structures considered in our dataset from each of the 8 families

| **Family** | **PDB_id** |
| --- | --- |
| Family1 | 1gu7, 1h2b, 1jqb, 1jvb, 1kol, 1pof, 1pl8, 1u3w, 2dm6, 2fzw, 2jhf |
| Family2 | 1cyd, 2c07, 1hdo, 1nff, 1ooe, 1sby, 1uay, 1uzm,1x1t, 2ag5, 2bd0, 2bka, 2fr1 |
| Family3 | 1dpg, 1dss, 1ff9, 1gad , 1lc0, 1mb4 , 1nvm, 1pqu, 1q0q, 1rm4, 1t4b, 1u8f, 1vkn, 2czc, 2g82, 2gz1, 3cmc |
| Family4 | 1dxy,1j4a, 1mx3, 1pjc, 2nac |
| Family5 | 1b8p, 1guz, 1hye, 1lld, 1mld, 1o6z, 1obb, 1pzg, 1t2d, 1uxj, 1y7t, 6ldh, 9ldt |
| Family6 | 1evy, 1f0y,1ks9, 1mv8, 1np3, 1txg, 1vpd, 1yqg, 2cvz, 2f1k, 2g5c, 2pgd, 2pv7 |
| Family7 | 1a4i, 1bgv, 1gpj, 1lua, 1npy, 1nyt, 1p77 |
| Family8 | 1id1, 1lnq, 1lss, 2fy8, 2hmt |

**Table S2**: (a-b) Secondary structural details of the residues forming the top 20 family wise conserved edges for one representative member of each family.

| **Top20 Edges** | **Family 1(1POF)** | | **Family 2(1OOE)** | | **Family 3(1Q0Q)** | | **Family4(1PJC)** | |
| --- | --- | --- | --- | --- | --- | --- | --- | --- |
|  | **Residue** | **S.S.Info** | **Residue** | **S.S.Info** | **Residue** | **S.S.Info** | **Residue** | **S.S.Info** |
| 141 151 | G35 G40 | L-H | G9 G15 | L-H | G8 G14 | L-H | G39 G44 | L-H |
| 147 151 | G37 G40 | L-H | G12 G15 | L-H | G11 G14 | L-H | G41 G44 | L-H |
| 143 194 | L36 G59 | L-L | K11 D33 | L-L | T10 A35 | L-L | G40 D62 | L-L |
| 151 155 | G40 A43 | H-H | G15 I18 | H-H | G14 T17 | H-H | G44 A47 | H-H |
| 136 414 | A32 V98 | L-E | I6 V72 | L-E | T5 V94 | L-E | V36 A96 | L-E |
| 133 424 | S29 D99 | L-L | G3 D73 | L-L | K2 D95 | L-E | G33 D97 | L-L |
| 429 561 | C104 L128 | L-L | V78 T127 | E-L | A100 A123 | L-L | G101 V131 | E-L |
| 151 429 | G40 C104 | L-H | G15 V78 | L-H | G14 A100 | L-H | G44 A102 | L-H |
| 137 151 | V33 G40 | E-H | V7 G15 | E-H | I6 G14 | E-H | I37 G44 | E-H |
| 141 431 | G35 A105 | L-L | G9 A79 | L-L | G8 I101 | L-L | G39 V103 | L-L |
| 135 425 | C31 Y100 | E-E(mid) | V5 G74 | E-E(mid) | L4 Q96 | E-E(mid) | V35 L98 | E-E(mid) |
| 134 186 | T30 R54 | E-E(term) | K4 T28 | E-E(term) | Q3 R29 | E-E(term) | K34 Q57 | E-E(term) |
| 139 193 | F34 V58 | E-E((term) | Y8 I32 | E-E(term) | L7 V34 | E-E(term) | L38 D62 | E-E(term) |
| 425 557 | Y100 V124 | E-E(term) | A76 L123 | E-E(mid) | Q96 T119 | E-E(term) | L98 V127 | E-E(term |
| 147 152 | G37 F41 | L-H | G12 G15 | L-H | G11 C15 | L-H | G41 T45 | L-H |
| 136 426 | A32 A101 | E-E(mid) | I6 V75 | E-E(mid) | T5 V97 | E-E(mid) | V36 L99 | E-E(mid |
| 150 429 | V39 C104 | L-H | L14 V78 | L-H | I13 A100 | L-H | V43 A102 | L-H |
| 428 560 | E103 V127 | E-E(term) | C77 L126 | E-E(term) | A99 L122 | E-E(term) | G101 D130 | E-E(term |
| 137 427 | V33 V102 | E-E(mid) | V7 F76 | E-E(mid) |  | E-E(mid) |  | E-E(mid) |
| 141 194 | G35 G59 | L-L | G9 D33 | L-L | G8 A35 | L-E | G39 D62 | L-E |

| **Top20 Edges** | **Family5(1LLD)** | | **Family6(2F1K)** | | **Family7(1GPJ)** | | **Family8(ILSS)** | |
| --- | --- | --- | --- | --- | --- | --- | --- | --- |
|  | **Residue** | **S.S.Info** | **Residue** | **S.S.Info** | **Residue** | **S.S.Info** | **Residue** | **S.S.Info** |
| 141 151 | G8 G13 | L-H | G7 G12 | L-H | G31 G36 | L-H | G7 G12 | L-H |
| 147 151 | G10 G13 | L-H | G9 G12 | L-H | G33 G36 | L-H | G9 G12 | L-H |
| 143 194 | A9 D33 | L-L | L8 S30 | L-L | A32 N55 | L-L | I8 D30 | L-L |
| 151 155 | G13 L16 | H-H | G12 L15 | H-H | G36 V39 | H-H | G12 L15 | H-H |
| 136 414 | A5 A70 | L-E | G4 A58 | L-E | L28 S85 | L-E | I4 A66 | L-E |
| 133 424 | T2 D71 | L-L | M1 K59 | L-L | K25 D86 | L-L | M1 D67 | L-L |
| 429 561 | T76 I117 | L-L | L64 V90 | L-L | A91 I122 | L-L | A71 R97 | E-E(term) |
| 151 429 | G13 T76 | L-H | G12 L64 | L-H | G36 A91 | L-H | G12 A71 | E-H |
| 137 151 | V6 G13 | E-H | V5 G12 | E-H | V29 G36 | E-H | I5 G12 | E-H |
| 141 431 | G8 A77 | L-L | G7 T65 | L-L | G31 T92 | L-L | G7 T73 | L-L |
| 135 425 | L4 M72 | E-E(mid) | I3 I61 | E-E(mid) | V27 V87 | L-L | I3 M68 | E-E(term) |
| 134 186 | K3 E28 | E-E(term) | K2 Y25 | E-E(term) | T26 A50 | E-E(mid) | Y2 D25 | L-L |
| 139 193 | I7 E34 | E-L | V6 V29 | E-E(term) | V30 A54 | E-E(term) | A6 I29 | E-E(term |
| 425 557 | M72 I113 | E-E(term | I61 I86 | E-E(mid) | V87 L118 | E-E(term) | M68 K93 | E-E(term) |
| 147 152 | G10 G13 | L-H | G9 A13 | L-H | G33 K37 | E-E(term) | G8 Y13 | L-H |
| 136 426 | A5 V73 | E-E(mid) | G4 I61 | E-E(mid) | L28 V88 | L-H | I4 Y69 | E-E(mid) |
| 150 429 | V12 T76 | L-H | I11 L64 | L-H | M35 A91 | E-E(mid) | V11 V72 | L-H |
| 428 560 | I75 L116 | E-E(term | L63 D89 | E-E(term) | S90 D121 | L-L | A71 A96 | E-E(term) |
| 137 427 | V6 V74 | E-E(mid) | V5 F62 | E-E(mid) | V29 M98 | E-E(term) | I5 I70 | E-E(mid) |
| 141 194 | G8 D33 | L-L | G7 S30 | L-L | G31 N55 | L-L | G7 D30 | E-L |

H, E and L stand for helix, β-sheets and loops respectively. E is further classified as mid and term representing middle and C-termini of the β-sheets respectively.

**Table S3**: Distribution of the constituting interactions/edges of the spatial structural motif across the 84 structures in our dataset.

| **Str. No.** | **141 151** | **147 151** | **143 194** | **151 155** | **429 561** | **151 429** | **137 151** | **141 431** |
| --- | --- | --- | --- | --- | --- | --- | --- | --- |
| Str1 | + | + | + | + | + | - | + | - |
| Str2 | + | - | + | - | + | + | - | + |
| Str3 | + | + | + | - | + | - | - | + |
| Str4 | + | - | + | + | + | - | + | + |
| Str5 | + | + | + | + | - | - | - | + |
| Str6 | + | + | + | + | - | + | + | + |
| Str7 | + | + | + | + | + | - | + | + |
| Str8 | + | + | + | + | + | + | + | + |
| Str9 | + | + | + | + | + | + | + | + |
| Str10 | + | + | + | - | + | - | - | + |
| Str11 | + | + | + | + | + | + | + | + |
| Str12 | - | + | - | + | + | + | - | + |
| Str13 | + | + | - | + | + | - | + | + |
| Str14 | + | + | + | + | + | - | + | - |
| Str15 | + | + | + | + | + | + | + | + |
| Str16 | + | + | + | + | - | + | + | + |
| Str17 | + | + | - | + | + | + | + | + |
| Str18 | + | - | + | - | - | + | - | - |
| Str19 | + | + | + | + | + | + | + | + |
| Str20 | + | + | + | + | - | + | + | + |
| Str21 | + | + | - | - | - | - | - | - |
| Str22 | + | + | + | + | - | + | + | + |
| Str23 | + | + | - | - | - | + | + | + |
| Str24 | + | + | + | + | + | + | + | - |
| Str25 | + | + | + | + | - | + | + | - |
| Str26 | + | + | - | - | - | - | + | + |
| Str27 | + | + | + | + | + | + | - | + |
| Str28 | - | + | - | + | + | + | + | + |
| Str29 | + | + | - | + | + | + | + | + |
| Str30 | + | + | + | + | + | - | - | + |
| Str31 | + | - | + | + | + | + | + | + |
| Str32 | + | + | - | + | + | + | + | - |
| Str33 | + | - | + | + | + | + | - | + |
| Str34 | + | - | + | + | - | + | + | - |
| Str35 | + | + | + | - | - | + | - | + |
| Str36 | + | + | - | + | - | + | + | + |
| Str37 | + | + | + | - | + | - | + | + |
| Str38 | - | + | + | - | + | + | - | - |
| Str39 | - | - | + | - | + | - | - | - |
| Str40 | + | + | + | + | + | + | + | + |
| Str41 | + | + | + | + | - | + | + | - |
| Str42 | + | + | + | - | + | - | + | + |
| Str43 | + | - | + | + | - | + | + | + |
| Str44 | + | + | + | + | + | - | + | + |
| Str45 | + | + | - | + | + | - | + | - |
| Str46 | + | + | + | + | + | - | + | - |
| Str47 | + | - | + | + | - | - | - | - |
| Str48 | + | + | - | + | + | - | + | - |
| Str49 | - | + | + | + | + | - | + | - |
| Str50 | + | + | + | + | + | - | - | - |
| Str51 | + | + | - | + | + | + | - | + |
| Str52 | - | + | - | + | + | - | + | - |
| Str53 | + | + | + | - | + | - | + | + |
| Str54 | + | + | + | + | + | + | + | + |
| Str55 | + | - | + | + | - | + | + | + |
| Str56 | + | + | + | + | + | - | + | + |
| Str57 | + | - | + | + | + | + | + | - |
| Str58 | + | + | + | + | - | - | + | - |
| Str59 | - | + | + | - | + | - | - | - |
| Str60 | + | - | + | + | + | + | + | + |
| Str61 | + | + | + | + | + | - | - | + |
| Str62 | + | - | + | - | - | - | + | + |
| Str63 | - | - | + | + | + | + | + | - |
| Str64 | + | - | + | + | + | + | + | + |
| Str65 | + | + | - | + | + | + | - | - |
| Str66 | + | - | + | + | + | + | + | + |
| Str67 | - | + | + | - | - | - | - | - |
| Str68 | - | - | - | - | - | + | - | - |
| Str69 | - | + | - | - | - | - | - | - |
| Str70 | + | + | - | + | + | + | + | + |
| Str71 | + | - | - | - | + | + | - | - |
| Str72 | + | + | - | - | + | - | + | + |
| Str73 | + | + | + | + | + | - | + | + |
| Str74 | + | - | + | + | + | + | + | + |
| Str75 | + | + | + | + | + | + | + | + |
| Str76 | + | + | + | + | + | - | - | - |
| Str77 | + | + | + | + | - | + | + | + |
| Str78 | + | + | + | + | + | + | + | + |
| Str79 | - | + | - | + | - | + | - | - |
| Str80 | + | - | + | + | + | + | + | + |
| Str81 | + | + | + | - | + | + | - | - |
| Str82 | + | + | + | + | + | + | + | + |
| Str83 | + | + | + | + | + | + | + | - |
| Str84 | - | + | + | + | - | - | - | - |

A +/- sign indicates that a particular interaction is present/absent in the corresponding structure respectively.

**Table S4: Overlap of the important residues in the context of the four network parameters (namely hubs, conserved edges, NB and EB) at I_min_ values of 2, 3 and 4%**

|  | **Imin_2** | **Imin_3** | **Imin_4** |
| --- | --- | --- | --- |
| Hubs | *136,137*,**139,141,143,151,** **152,155,156,158,159,191, 392,**399*,425,426*,**427,428 ,** **515,531**,*536*,**557,558,560**  **561,626,658,660** | *136,137,***139,141,143,151,**  **152,155,156,158,159,191,**  **392,***425,426,***427,428, 558** **515,531,***536,***557, 660, 560,**  **561,626,658** | **139,141,143,151**,**152, 155,**  **156, 157,158,191,392,427**  **428,515,531**,**557,558,560**  **561,626,658,660** |
| Edges | **133,134,135**,*136*,**137,139**,  **143,147,150,151,152,155,**  **186,193,194***,414*,**424,425 ,**  **426,427,428,429,**431,557,  560,561 | **133,134,135**,*136*,**137,139,**  **143,147**,**150,151,152,155**  **186**,**193,194,**414,**424,425,**  **426,427,428,429** | **133,134,135,137,139,143**  **147,150,151,152,155,186**  **186,193,194,424,425**  **426,427,428,429** |
| NB | 133,134, *135*,**136,137,139,**  **141,151,155,156,159,191 193,392**,*424,***425,426,427,**  **428,429***,431*,508,**515,531**,  557,**558,559,560,626,660** | *135*,**136,137,139,141**,**151**,  **155,156,159**,**191,193**,**392**,  *424*,**425,426427,428,429,**  *431***,515,531**,**558,559,**  **560, 626,660** | **136,137,139,141,151,155,156,**  **159,191,193,392,427,428,429,**  **515,531,558,559,560,**  **626,660** |
| EB | 133,**134,135,136***,137*,**139,**  **141,147**,150,151**,155,156,**  **159**,186**,191,193, 194,414** 424*,425,***426,427, 428,429**  **515,***557,***559** | **134,135,136,***137***,139,141**  **143,147,150,151,155,156,**  **159,193,194,414*,****425,***426**  **427,428,429,515,***557*,**559** | **134,135,136,139,141,**  **147,150,151,155,156,159,**  **191,193,194,414,426,427,**  **428,429, 515,559.** |

The residues that are common for all the three Imin values are shown in bold, and those common between Imin 2 and 3% are in italics. Such a representation exhibits that results at 4% is a subset of 2 and 3% and the results at 3% are a subset of 2%.

**Table S5:** Comparison of the ‘fold-specific hot spots’ at Imin values of 2, 3, and 4%

| **Imin_2** | **Imin_3** | **Imin_4** |
| --- | --- | --- |
| 139  141  143  151  155  156  193  427  428  429  515  531  560  135  136  425  426  557  159  -  -  *424*  *536*  *558*  *133*  *134* | 139  141  143  151  155  156  193  427  428  429  515  531  560  135  135  425  426  557  -  191  559  *414* | 139  141  143  151  155  156  193  427  428  429  515  531  560  -  -  -  -  -  159  191  559  *561*  *147*  *194*  *261* |

The ones that are exclusive at each Imin are listed at the end in italicized fonts.

**Table S6**: Comparison between the top 25 “fold-specific hot spots” and the residues that participate in clique formation.

| **S. No** | **Positions/residues**  **forming cliques** | **Top 25**  **“fold-specific positions”** | **Common Positions** |
| --- | --- | --- | --- |
| 1 | 135 | 135 | 135 |
| 2 | 136 | 136 | 136 |
| 3 | 137 | 137 | 137 |
| 4 | 139 | 139 | 139 |
| 5 | 141 | 141 | 141 |
| 6 | 143 | 143 | 143 |
| 7 | 151 | 151 | 151 |
| 8 | 155 | 155 | 155 |
| 9 | 156 | 156 | 156 |
| 10 | 159 | 159 | 159 |
| 11 | 193 | 193 | 193 |
| 12 | 425 | 425 | 425 |
| 13 | 426 | 426 | 426 |
| 14 | 427 | 427 | 427 |
| 15 | 428 | 428 | 428 |
| 16 | 429 | 429 | 429 |
| 17 | 515 | 515 | 515 |
| 18 | 531 | 531 | 531 |
| 19 | 558 | 558 | 558 |
| 20 | 560 | 560 | 560 |
| 21 | 392 | 536 |  |
| 22 | 532 | 557 |  |
| 23 | 557 | 134 |  |
| 24 | 561 | 424 |  |
| 25 | 619 |  |  |
| 26 | 662 |  |  |
| 27 | 189 |  |  |
| 28 | 190 |  |  |
| 29 | 191 |  |  |

80% of the top 25 “fold-specific residues” coincide with those participating in clique formation (i.e. higher order connectivity).

**Table S7**: Maximum sequence identity (similarity) of each member of our dataset with the rest of the dataset.

| **Max. Sequence Identity (similarity)**  **of each structure in the dataset** |
| --- |
| 32.2% identity (62.7% similar)  71.6% identity (88.4% similar)  ----  33.2% identity (61.9% similar  26.7% identity (53.3% similar)  69.3% identity (90.2% similar)  40.4% identity (71.4% similar)  25.6% identity (61.4% similar)  30.2% identity (60.5% similar)  25.8% identity (60.2% similar)  58.4% identity (81.3% similar)  27.9% identity (69.1% similar)  23.5% identity (56.1% similar)  55.3% identity (80.3% similar)  38.3% identity (73.1% similar)  27.8% identity (58.2% similar)  34.9% identity (61.7% similar)  21.6% identity (66.4% similar)  40.7% identity (70.9% similar)  31.2% identity (62.3% similar)  38.3% identity (73.1% similar)  35.6% identity (66.3% similar)  37.9% identity (69.0% similar)  ----  43.9% identity (79.1% similar)  21.6% identity (66.4% similar)  25.6% identity (65.0% similar)  32.7% identity (60.4% similar)  69.3% identity (86.4% similar)  34.9% identity (61.7% similar)  35.0% identity (65.0% similar)  34.6% identity (60.6% similar)  38.6% identity (66.4% similar)  35.2% identity (64.8% similar)  23.6% identity (62.4% similar)  ----  47.1% identity (77.1% similar)  35.2% identity (66.2% similar)  29.5% identity (67.2% similar)  24.5% identity (56.9% similar)  60.2% identity (84.1% similar)  47.1% identity (77.1% similar)  31.9% identity (51.7% similar)  26.3% identity (63.5% similar)  72.6% identity (89.7% similar)  55.4% identity (83.8% similar)  ----  51.5% identity (78.9% similar)  29.2% identity (56.2% similar)  55.4% identity (83.8% similar)  72.6% identity (89.7% similar)  25.6% identity (61.4% similar)  89.1% identity (97.7% similar)  69.3% identity (90.2% similar)  31.6% identity (57.0% similar)  55.3% identity (80.3% similar)  34.9% identity (68.5% similar)  28.4% identity (58.1% similar)  36.1% identity (60.0% similar)  38.6% identity (66.4% similar)  27.6% identity (62.1% similar)  27.8% identity (58.2% similar)  31.1% identity (59.4% similar)  24.7% identity (60.9% similar)  27.9% identity (69.1% similar)  32.5% identity (65.0% similar)  36.1% identity (60.0% similar)  ----  29.9% identity (56.7% similar)  33.3% identity (61.6% similar)  ----  99.2% identity (100.0% similar)  68.0% identity (91.4% similar)  33.3% identity (61.0% similar)  59.2% identity (81.1% similar)  28.4% identity (58.1% similar)  25.6% identity (65.0% similar)  89.1% identity (97.7% similar)  34.6% identity (60.6% similar)  27.7% identity (54.1% similar)  31.4% identity (59.3% similar)  59.2% identity (81.1% similar)  81.9% identity (93.8% similar)  81.9% identity (94.4% similar) |

The average sequence identity is less than 40% (±20%) allowing us to focus our analyses only on non-homologous sequences. The “----” sign indicates that the identity of this member is insignificant with the rest of the dataset.
